# Integration of immune cells in organs-on-chips: a tutorial

**DOI:** 10.3389/fbioe.2023.1191104

**Published:** 2023-06-01

**Authors:** Lisette Van Os, Britta Engelhardt, Olivier T. Guenat

**Affiliations:** ^1^ Organs-on-Chip Technologies, ARTORG Center for Biomedical Engineering, University of Bern, Bern, Switzerland; ^2^ Graduate School for Cellular and Biomedical Sciences, University of Bern, Bern, Switzerland; ^3^ Theodor Kocher Institute, University of Bern, Bern, Switzerland; ^4^ Department of Pulmonary Medicine, Inselspital, University Hospital of Bern, Bern, Switzerland; ^5^ Department of General Thoracic Surgery, Inselspital, University Hospital of Bern, Bern, Switzerland

**Keywords:** infection, organ-on-chip, infection-on-chip, endothelium, immune cells, hydrogel ECM, chemoattractant, inflammation

## Abstract

Viral and bacterial infections continue to pose significant challenges for numerous individuals globally. To develop novel therapies to combat infections, more insight into the actions of the human innate and adaptive immune system during infection is necessary. Human *in vitro* models, such as organs-on-chip (OOC) models, have proven to be a valuable addition to the tissue modeling toolbox. The incorporation of an immune component is needed to bring OOC models to the next level and enable them to mimic complex biological responses. The immune system affects many (patho)physiological processes in the human body, such as those taking place during an infection. This tutorial review introduces the reader to the building blocks of an OOC model of acute infection to investigate recruitment of circulating immune cells into the infected tissue. The multi-step extravasation cascade *in vivo* is described, followed by an in-depth guide on how to model this process on a chip. Next to chip design, creation of a chemotactic gradient and incorporation of endothelial, epithelial, and immune cells, the review focuses on the hydrogel extracellular matrix (ECM) to accurately model the interstitial space through which extravasated immune cells migrate towards the site of infection. Overall, this tutorial review is a practical guide for developing an OOC model of immune cell migration from the blood into the interstitial space during infection.

## Introduction

The COVID-19 pandemic has highlighted the ongoing danger posed by pathogens or microbes to our society. New disruptive research is necessary to gain a deeper understanding on how these pathogens infect specific cells in our body and the tissue damage they inflict, and to develop methods to mitigate and prevent their spread. Organs-on-chip (OOC) are microphysiological models aiming to replicate an organ’s functional unit. Over the past decade, the OOC field has experienced exponential development (Ingber, 2022). Since the first lung-on-chip was published in 2010 (Huh et al., 2010), many other OOCs and multi-organ-on-chip models have been developed. Although advances in the OOC field have been remarkable, the inclusion of cellular immune components in these systems is a recent development. The immune system plays a significant role in organ homeostasis and the inflammatory tissue response upon infection. Therefore, pathologies modeled in OOC systems without the respective cellular immune component will deviate significantly from human physiology.

Multiple reviews have summarized immunocompetent OOC models (Maharjan et al., 2020; Miller et al., 2020; Morsink et al., 2020) and the interactions between pathogens and the immune system on chip (Tang et al., 2020; Feaugas and Sauvonnet, 2021; Saygili et al., 2021). While these reviews excellently describe the state-of-the-art, the practical aspects of infection-on-chip research are not discussed in-depth.

This tutorial review aims at providing a guide for both experts and newcomers in the field on establishing an immunocompetent OOC model of the inflammatory process during infection, either by modelling inflammation with a chemoattractant or by modelling infection with a pathogen. It includes an introduction to the innate and adaptive immune system, with a focus on recruitment of innate immune cells, followed by key aspects to consider when modelling infection on a chip. Special focus is provided on integrating an endothelial and epithelial barrier on a chip using a hydrogel ECM, as well as important experimental aspects to be considered. The review also discusses incorporating immunity on-chip, mimicking infection, monitoring immune cell transmigration across the vascular wall and within the tissue and presents future perspectives on the next level of immunocompetent OOC systems.

## Immune cell extravasation cascade *in vivo*

The inflammatory cascade involves a complex interplay of various immune cells and signaling molecules interacting with non-immune cells, such as epithelial and endothelial cells. Tissue resident immune cells, the first to be activated by pathogens, release cytokines that activate the local blood vascular endothelium. The activated blood vascular endothelium increases its expression of adhesion molecules and chemokines, which attracts circulating immune cells (Maas et al., 2018). The immune cells transmigrate through the endothelial layer, cross the underlying basement membrane, and migrate through the tissue towards the site of infection (Abbas et al., 2012). More exhaustive descriptions of the mechanisms and pathways involved in immune cell transmigration can be found in other reviews (Ley et al., 2007; Abbas et al., 2012; Sturtzel et al., 2017; Maas et al., 2018).

It is essential to distinguish between the innate and adaptive immune response: the innate immune response is the first, fast response to infection, whereas the adaptive immune response is pathogen-specific and generates memory cells for resolving future infections with the same pathogen (Chaplin, 2010; Abbas et al., 2012). For an effective adaptive immune response, incorporation of the lymphatics is necessary, to allow for dendritic cell migration from the site of infection to the lymph nodes, presenting antigens to T Cells in the lymph nodes. This review will focus on the innate immune response, where innate immune cells, mainly neutrophils and monocytes, are recruited to the site of infection from the blood vasculature.

### Pathogen detection and innate immune response

**FIGURE 1:**
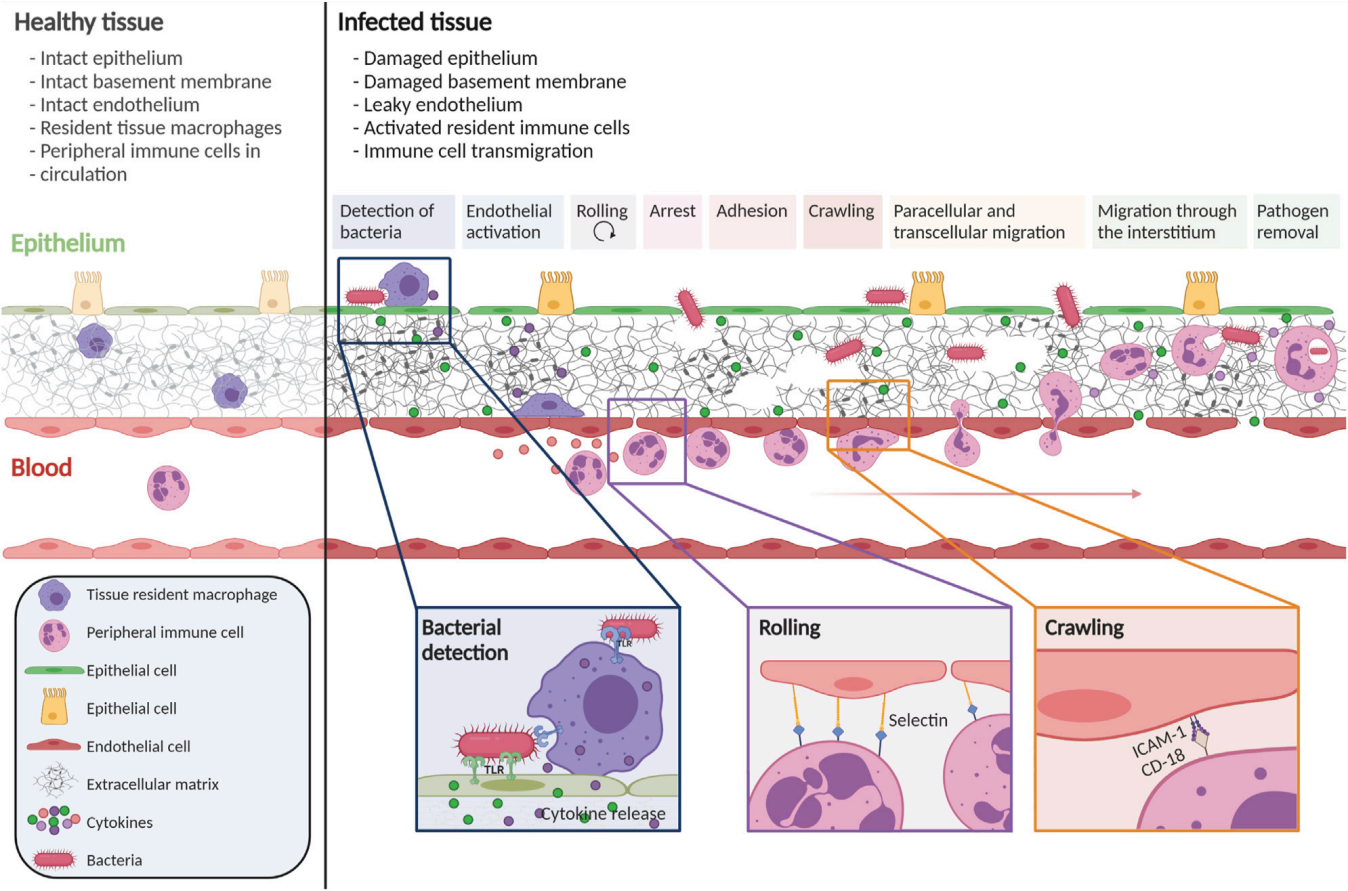
Immune cell transmigration cascade upon infection. The immune cell transmigration cascade is a multistep process. During infection, gaps in the epithelium, endothelium, and ECM are observed. Tissue-resident immune cells detect the pathogen and secrete cytokines to activate the endothelium and attract peripheral immune cells. Through multiple steps, peripheral immune cells are attracted towards the site of infection. This figure is based on numerous reviews and research publications (Ley et al., 2007; Abbas et al., 2012; Sturtzel et al., 2017; Alper et al., 2018; Maas et al., 2018). Created with BioRender.com.

Pathogens are typically detected by their pathogen-associated molecular patterns (PAMPs) (Alper et al., 2018), which, in turn, are recognized by pattern recognition receptors (PRRs) on cells in tissues. PRR activation induces an intracellular signaling cascade that triggers pro-inflammatory actions, such as cytokine production, to attract immune cells and activate blood vessel endothelial cells (BECs). The activated endothelium attracts peripheral immune cells, which transmigrate from the blood vessel to the infection site. This transmigration cascade is a multistep process (Figure 1), which includes the capture, rolling, arrest, crawling, and transmigration of immune cells at the level of post-capillary venules (Ley et al., 2007; Abbas et al., 2012; Sturtzel et al., 2017). After crossing the microvascular endothelium, immune cells migrate through the extracellular tissue space to the site of infection.

To initiate the process of capture and rolling, passively flowing immune cells must come into contact with the endothelium, which occurs when BECs are activated. BEC activation arises through cytokine signaling but also through altered blood flow. In normal laminar flow conditions, the BECs are tightly aligned, and the cells are in a quiescent state. However, when the flow is disturbed and oscillatory, BECs are less aligned because they sense the flow variations, leading to BEC activation (Heo et al., 2011). In both disturbed flow and cytokine activation, activated BECs upregulate adhesion molecules like E- and P-selectins and members of the IgCAM family such as ICAM-1 amd VCAM-1, the main molecules involved in immune cell capture and rolling (Ley et al., 2007; Abbas et al., 2012; Muller, 2013; Maas et al., 2018).

When the immune cells arrest after rolling, the cells flatten to decrease their exposure to shear stress and other circulating cells (Figure 1). (Maas et al., 2018) Chemoattractant exposure, as well as binding to integrins, is essential in regulating arrest with different signals inducing firmer adhesion of the immune cell to the endothelium. Firm adhesion to the endothelium allows the cells to crawl along the endothelium to find the optimal location for transmigration across the endothelial cell wall. The majority of cells follow the paracellular transmigration route, where the cell migrates between BECs, via the cell-to-cell junctions (Muller, 2011). A smaller percentage of immune cells opt for transcellular diapedesis, in which the immune cell crosses the barrier by going through a BEC.

### Immune cell migration through the interstitium

After reaching the extracellular tissue space, the immune cells’ ability to migrate towards the site of infection is crucial. To accomplish this, the cell tracks various chemokines through the dense extracellular matrix (ECM) network. Tissue-resident macrophages and the epithelium produce chemokines to stimulate migration. To ensure that chemokines are correctly positioned, they are bound to the ECM, often to proteoglycans in the ECM (Gill et al., 2010; O’Dwyer et al., 2018). Immune cells respond to chemokines based on factors such as chemoattractant type, concentration, and exposure time (Amulic et al., 2012). Resident cells interact with the migrating immune cells and can also produce matrix-modifying enzymes, such as matrix metalloproteinases (MMPs) and collagenases, which specifically degrade the ECM, thus potentially facilitating movement of immune cells through the tissue (Parks et al., 2004; O’Dwyer et al., 2018; Miskolci et al., 2021). The combined action of MMP production, chemokine signaling, and cell-cell interaction facilitates immune cell migration towards the site of infection in the extracellular tissue space.

### *In vivo* models of immune cell extravasation

*In vivo* studies on innate immune cell extravasation across the vascular wall have primarily focused on easily accessible structures, such as muscle, skin, and mesentery (Halin et al., 2005). Research on these tissues benefits from better imaging quality than internal tissues that are challenging to reach. Within these tissues, immune cells mostly transmigrate from the postcapillary venule (Hickey and Westhorpe, 2013; Weirather and Frantz, 2015). The specific transmigration mechanisms used by immune cells vary considerably depending on the organ of interest, the immune cell subset, and the time during inflammation caused by infection. For example, in the skin, immune cells migrate from the postcapillary venule through a large extracellular space to reach the site of infection, whereas in the lung, the air-blood barrier is very narrow, resulting in the immune cells mostly transmigrating from the capillaries into the airspace (Maas et al., 2018). Thus, the molecular mechanisms by which immune cells transmigrate depend on their microenvironment, specifically the vascular characteristics of the respective tissue. Unfortunately, live imaging of extravasation in internal organ vessels can only be carried out after a complex surgical procedure or with expensive equipment such as a two-photon microscope to perform intravital imaging (Kim et al., 2019a). Surgery not only causes stress for the animals, which may activate immune cells, but anesthesia also influences vascular dilation and immune cell activation, making it crucial to identify alternative methods to study the mechanisms of inflammation and infection *in vitro* (Hickey and Westhorpe, 2013).

## *In vitro* models of immune cell extravasation

Two main types of *in vitro* models for immune cell extravasation are commonly used: (1) static models and (2) basic flow chambers (Muller and Luscinskas, 2008; Salminen et al., 2020). In these models, a monolayer of BECs is cultured to confluency and immune cells are introduced either statically or under flow conditions. To generate an innate immune response, BECs and/or innate immune cells are exposed to a pro-inflammatory stimulus. In contrast, adaptive immune cells inherently extravasate, but a pro-inflammatory stimulus is necessary for activation of adaptive immune cells. The process of immune cell extravasation is visualized and analyzed using microscopy.

Static models consist of two compartments between which immune cells migrate by crossing the endothelium and then entering the second compartment. The most well-known model is the two-chamber migration assay, also known as the transwell assay (Falasca et al., 2011). In this assay, BECs are grown in a filter insert, and immune cells cross the endothelium and then squeeze through the pores of the filter insert to reach the lower compartment. Unfortunately, this system does not incorporate an ECM environment for immune cells to migrate into. A more elaborate assay, the collagen hydrogel transendothelial migration assay, was developed to address this issue. In this modified assay, an endothelial monolayer is formed on a collagen hydrogel, and immune cells migrate from the media space across the endothelial monolayer into the underlying hydrogel (Muller and Luscinskas, 2008).

In the bloodstream, immune cells are exposed to shear stress, affecting BEC function and immune cell transmigration (Brooks et al., 2002). To investigate this further, laminar flow chamber assays have been utilized to study immune cell adhesion and transmigration under flow conditions, allowing a better understanding of the extravasation cascade and the role of selectins in adhesion and rolling (Salminen et al., 2020). However, these assays involve culturing BECs on stiff and rigid substrates without an ECM hydrogel to simulate the soft interstitial space. To address this limitation, OOC models offer a more advanced platform, allowing for the co-culture of multiple cell types, including physiological fluid flow, and cell culture on soft hydrogel ECMs.

## Immune cell extravasation in organs-on-chip

**FIGURE 2:**
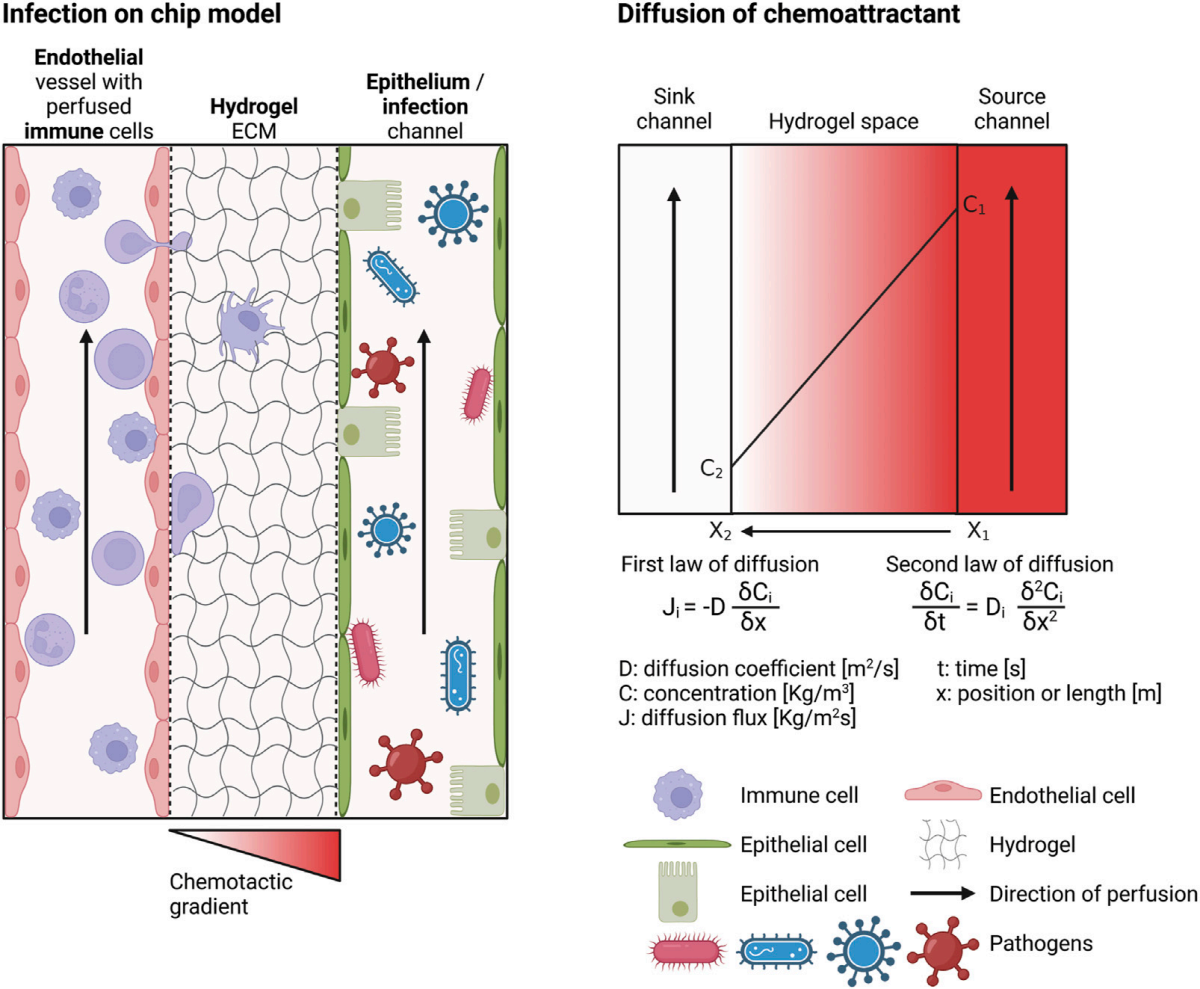
Requirements of an infection-on-chip model. The infection-on-chip schematic shows the main elements needed to create an infection-on-chip model: microvascular endothelial cells, epithelial cells, immune cells (optimally peripheral immune cells in the blood vessel and tissue resident immune cells in the ECM), a hydrogel mimicking the ECM environment, and a chemotactic gradient to induce inflammation or a pathogen to model infection. The chemoattractant diffusion is depicted (right side). Created with BioRender.com.

In the following sections, the development of an OOC model of infection (infection-on-chip) will be discussed. Individual components of the model are shown in Figure 2: the chip design, hydrogel ECM, microvascular endothelium, epithelium, tissue resident and circulating immune cells, and induction of inflammation with a chemotactic gradient or mimicking infection with a pathogen. These components, with practical aspects on how to establish them in the laboratory, will be elaborated on later in this review.

### Microfluidic chip design

**FIGURE 3:**
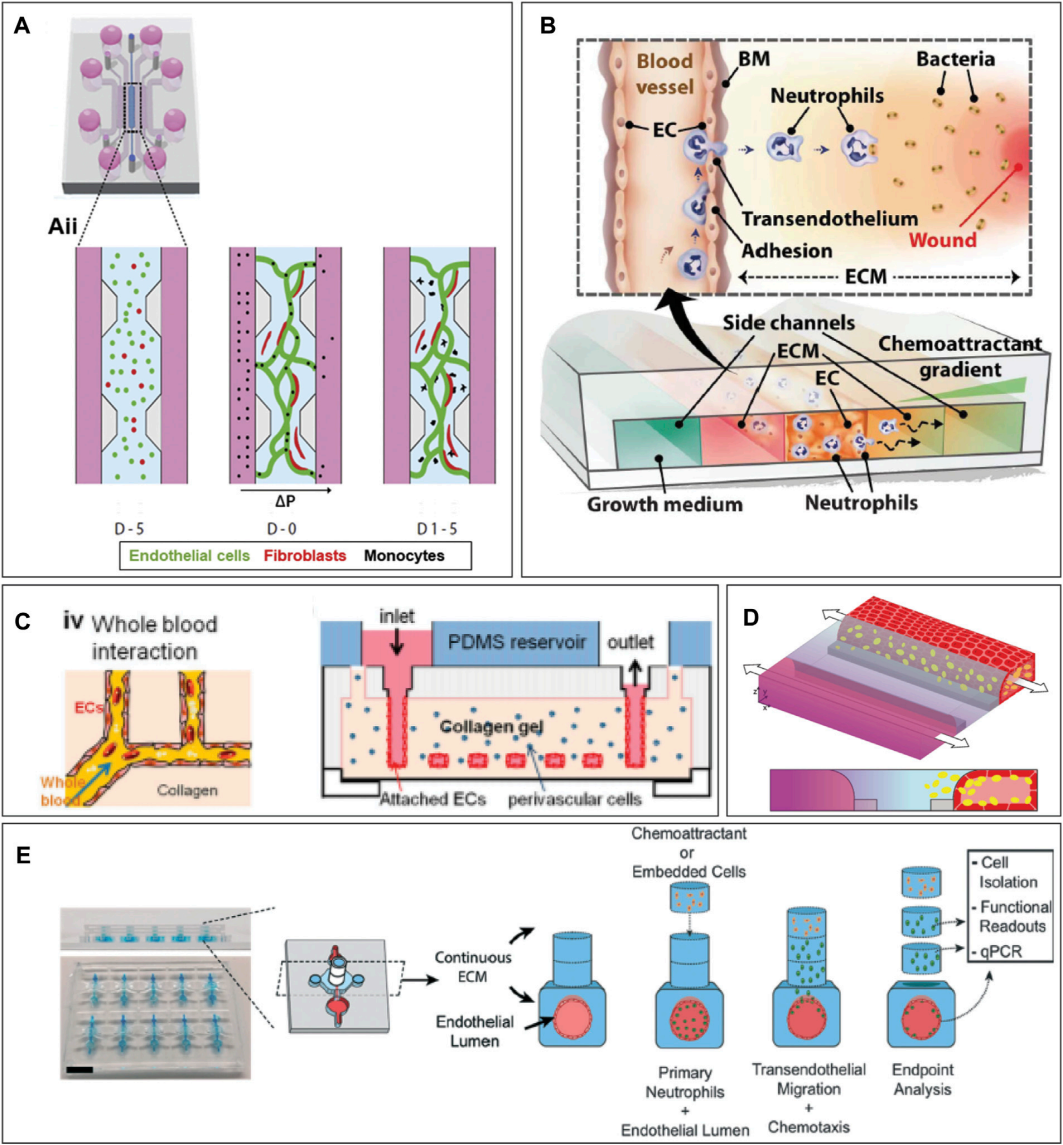
Examples of organ-on-chip designs incorporating hydrogels. **(A)** Pillar-based system for microvasculature formation. Image from Boussommier et al. (Boussommier-Calleja et al., 2019) **(B)** Neutrophil extravasation system. Image from Han et al. (Han et al., 2012) **(C)** Hydrogel-based chip with molded vessel structure within PDMS device. Image from Zheng et al. (Zheng et al., 2012) **(D)** A phaseguide separates the hydrogel from the media channel, creating a single blood vessel in the media channel. **(E)** Neutrophil extravasation on chip using stacks. Image from McMinn et al. (McMinn et al., 2019). Figure D was adapted from De Haan et al. (de Haan et al., 2021), under the terms of the Creative Commons Attribution License (CC BY). (https://creativecommons.org/licenses/by/4.0/).

A microfluidic chip to model immune cell extravasation and migration during infection should be multicompartmental, to enable the creation of an endothelium-hydrogel-epithelium barrier, and perfusable, to perfuse immune cells and create a chemotactic gradient (Figure 2). Several commercialized microfluidic chips are available, such as from Mimetas, AimBiotech, and BEOnChip (Zhang and Radisic, 2017; Zommiti et al., 2022). However, a customized microfluidic chip can be developed if a specific design is desired. Figure 3 gives an overview of possible chip designs. Information concerning chip fabrication, typically using soft lithography, can be found elsewhere. (Cameron et al., 2022; Cho et al., 2022; Leung et al., 2022). Briefly, a mold is created by 3D printing or stereolithography and filled with polydimethylsiloxane (PDMS) mixed with a curing agent. Cured PDMS is post-processed and bonded to another PDMS part or a glass slide to close channel structures. The chip’s design is based on the abovementioned requirements: multi-compartmentalization with a hydrogel ECM and perfusability. Generally, designs can be categorized into two groups: chips with channels created inside the hydrogel structure and chips with a hydrogel barrier separating the microfluidic channels.

#### Blood vessels created in hydrogel chips

In hydrogel-based chips, the microfluidic channels are created into the hydrogel itself, and structural material, like PDMS, is only utilized to encapsulate the structure for easier handling. To create channels inside a hydrogel, a mold can be generated out of a substrate such as PDMS, and the hydrogel is polymerized on top of the mold to create the channels (Figure 3C). (Zheng et al., 2012) These channels can be used to assess bacterial and mammalian cell chemotaxis (Cheng et al., 2007) and microvessels can be molded (Qiu et al., 2018). To create a single cylindrical blood vessel, a hydrogel is formed with a removable needle inside (Park et al., 2010; Tourovskaia et al., 2014; Zeinali et al., 2021). Such devices can be used to study immune cell extravasation from the blood vessel lumen into the hydrogel (Lee et al., 2017), to create multiple vessels inside a hydrogel with different BEC types (Hasan et al., 2015), and create hydrogel stacks on top of the blood vessel to investigate neutrophil migration distance (Figure 3E). (McMinn et al., 2019) Moreover, the hydrogel surrounding the vessel can be dried to create a stable, dense collagen construct in which endothelial cells can be grown and neutrophil extravasation can be observed (Chen et al., 2018). A sacrificial hydrogel can be used to create structures within different hydrogels. For example, agarose can be encapsulated by crosslinked gelatin hydrogels. After gelatin gelation, the agarose can be flushed out, and a cylindrical lumen remains (Bertassoni et al., 2014). Lastly, structures inside a hydrogel can be formed through laser ablation. With this method, a laser specifically ablates the regions of interest to create a unique microchannel design inside a hydrogel on chip (Brandenberg et al., 2016; Nikolaev et al., 2020).

#### Microfluidic chips with hydrogel barrier compartment

To confine the hydrogel in one compartment, one can make use of surface tension. The hydrogel can be restricted within one channel using pillar structures (Huang et al., 2009; Bichsel et al., 2015; Adriani et al., 2017; Aizel et al., 2017; Campisi et al., 2018; Li et al., 2018; Zeinali et al., 2018; Boussommier-Calleja et al., 2019) (Figure 3A) or a channel height difference (Vulto et al., 2011; Poussin et al., 2020; de Haan et al., 2021; Riddle et al., 2022) (Figure 3D). This allows the user to create an endothelial and epithelial barrier alongside the hydrogel and observe immune cell migration through the hydrogel (Poussin et al., 2020; de Haan et al., 2021; Riddle et al., 2022). In another model, migration between the media and the gel channel is only possible via a number of smaller openings in the center of the chip (Shin et al., 2012). This model has been used to examine breast cancer metastasis as well as neutrophil transendothelial migration into the collagen hydrogel (Figure 3B). (Han et al., 2012; Bersini et al., 2014; Na et al., 2017) Lastly, a model with a suspended hydrogel can be created, for example, to model an airway-on-chip (Humayun et al., 2018).

Most designs discussed here currently do not include pump-assisted uni-directional flow. To include a pump-assisted flow of immune cells, a chip has to be adapted to be connected to a pump. This has been accomplished to investigate immune cell migration across an endothelial layer on chip (Zhang et al., 2016; Menon et al., 2017). Where one system focuses on forming a single channel with the aid of a collagen hydrogel (Menon et al., 2017), the other system engineers a biodegradable scaffold for a microvascular network from which immune cells can migrate (Zhang et al., 2016).

#### Experimental aspects of the chip design

With the aid of surface tension, a hydrogel can be contained in one microfluidic compartment of the chip. During the design phase, it is crucial to calculate the change in contact angle necessary to maintain the hydrogel in the correct compartment. These calculations, based on the Young–Laplace equation, have been extensively described (Huang et al., 2009).

A tight connection between the pipette tip and the hydrogel inlet is recommended to simplify pipetting the hydrogel into the chip. Ensuring this tight connection involves designing the inlet size with precise dimensions matching the end of the pipette tip.

The PDMS chip material is known for its poor cellular adherence (Leung et al., 2022). Hence, surface modification of the material to allow for cell attachment is essential. Usually, the material is coated with an ECM mixture, such as collagen-fibronectin (Dabaghi et al., 2021). The hydrophobicity of PDMS results in poor attachment of ECM proteins unless further modifications are made. Thus, PDMS pre-treatment with oxygen plasma or chemicals like polydopamine or (3-aminopropyl)triethoxy silane (APTES) is recommended (Dabaghi et al., 2021). Adding a pre-treatment with ECM coating improves BEC and hydrogel adhesion to the OOC, preventing cell detachment and hydrogel contraction.

### Hydrogels used to model the ECM environment

Hydrogels, as their name suggests, mainly consist of water, with a 3D polymer network forming the structure. The wide variety of available polymers with different properties enables the recapitulation of different ECM types (Liu et al., 2019). Importantly, a hydrogel’s mechanical properties can be tuned through various methods to be similar to those of human tissues. Hydrogel chemical composition and topographies can be adjusted to mimic the tissue of interest, and specific ECM components can be included if desired. An organ-specific hydrogel resembles the instructive native environment of the cells, to emulate a functional tissue environment.

The most commonly used hydrogels for on-chip culture are created from collagen, fibrinogen, Matrigel, gelatin, polyacrylamide (PA), polyethylene glycol (PEG), or hyaluronic acid (HA) (Caliari and Burdick, 2016). More recently, hydrogels comprising native tissue ECM have also been created to recapitulate the ECM of the organ of interest more accurately (Marhuenda et al., 2022). In most of the infection-on-chip models, hydrogels are the central environment between the epithelium and endothelium (Figure 2).

#### Effects of hydrogel properties on immune cell migration

**FIGURE 4:**
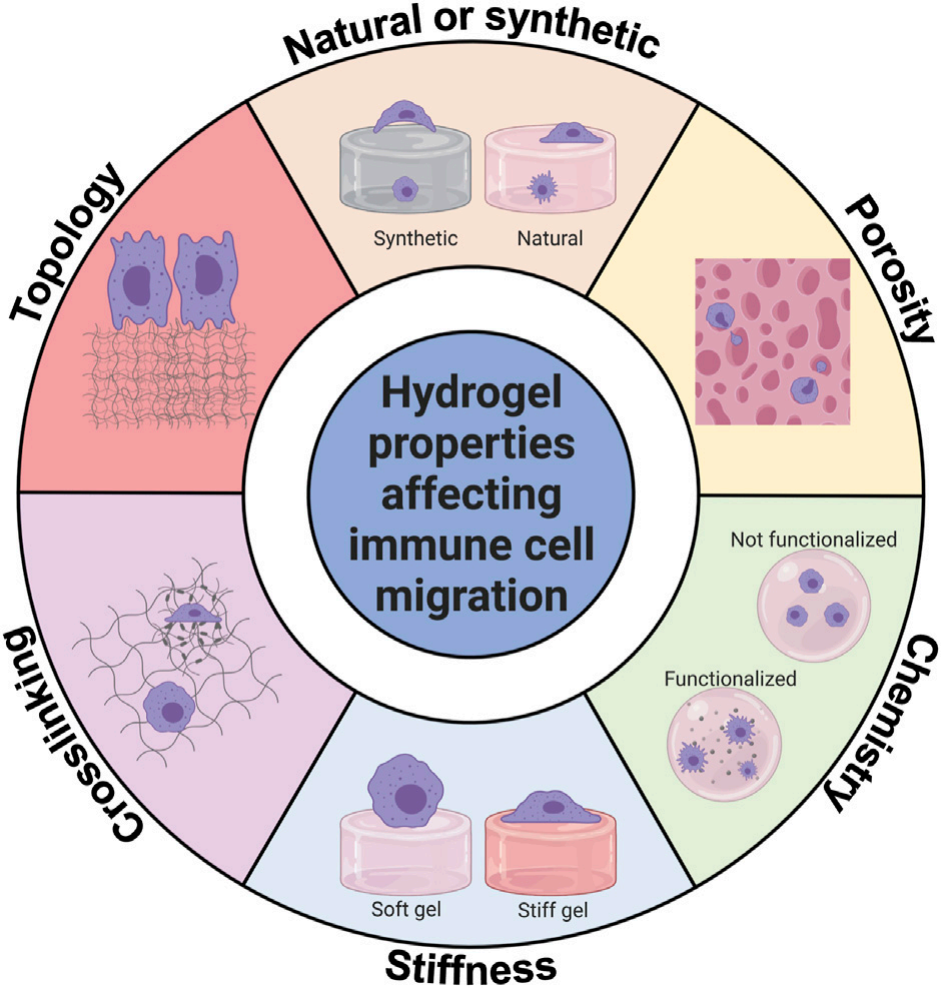
Hydrogel properties affecting innate immune cell migration. Innate immune cell migration is affected by hydrogel chemistry, type, stiffness, porosity, topology, and crosslinking. This figure shows graphically how innate immune cells can be affected. Created with BioRender.com.

Immune cells, like other cells, interact with the proteins in hydrogels. On top of a 2D hydrogel surface, the cells are less constrained. On the other hand, encapsulated inside a 3D hydrogel, the cells are more constrained but have more points of adhesion to the matrix which can be used for immune cell migration, but are not necessary for immune cells to migrate (Caliari and Burdick, 2016). Figure 4 gives an overview of the hydrogel properties that influence immune cell migration.

##### Natural and synthetic hydrogels

Synthetic hydrogels are created from non-natural polymers, such as polyacrylamide (PA) or polyethylene glycol (PEG), while naturally derived hydrogels are produced from, for example, collagen, fibrinogen, or gelatin (Lee et al., 2017; Liu et al., 2019). While naturally derived hydrogels are considered closest to the composition of native ECM, they are susceptible to biodegradation and contraction, leading to the deformation of the intended structure (Liu et al., 2019). In contrast, synthetic hydrogels are often bioinert, meaning they do not interact with immune cells unless activated by adding specific peptides to these hydrogels.

##### Hybrid hydrogels

Hybrid hydrogels are created by mixing different hydrogels, for example, by mixing one natural hydrogel with another, such as collagen and fibrinogen (Sano et al., 2018). A hybrid hydrogel, consisting of a mixture of Geltrex and collagen, was used to investigate neutrophil extravasation (Riddle et al., 2022). Results showed that neutrophils extravasated to a larger extent in geltrex/collagen mix gel compared to collagen only, exemplifying the effect of hydrogel composition on neutrophil migration.

##### Hydrogel chemistry

The functionalization of hydrogels with specific peptides can improve adhesion and alter cell behavior. This has been mainly observed with PEG hydrogels and the RGD peptide, a sequence of the three amino acids arginine, glycine, and aspartate. For example, implanting PEG alone induced a foreign body reaction with macrophage accumulation, which did not occur upon PEG-RGD conjugated hydrogel implantation (Lynn et al., 2010). On the other hand, naturally-derived hydrogels suffer from batch-to-batch variability, and their chemical structure can have unwanted effects on immune cells. For instance, fibrin is essential in inflammation and wound healing. *In vitro*, fibrin retains its chemotactic properties, attracting macrophages to the area where fibrin is present (Tanaka et al., 2019), which could influence experimental results.

##### Hydrogel porosity

Hydrogel porosity affects the mechanical structure and oxygen and nutrient diffusion. Collagen hydrogel density can be modified by altering collagen concentration in the hydrogel, which affects macrophage migration. In a dense collagen gel, macrophages migrate less than in a highly porous collagen gel (Ford et al., 2019; Pérez-Rodríguez et al., 2022). Another method of controlling density is by integrating soluble particles into a hydrogel, which, upon dissolution, leaves pores with a specific size (Lee et al., 2017). This method can be used to control pore size accurately.

##### Stiffness

Substrate stiffness is a crucial factor influencing immune cell migration. Hydrogels generally have low stiffness, which can be fine-tuned, making them optimal for assessing cell migration on different stiffness substrates. To create stiffness gradients, most studies adapt the concentration of hydrogel molecules or utilize a crosslinker (Whang and Kim, 2016).

On stiffer hydrogels, neutrophils flatten and spread more than on soft hydrogels, where they remain rounded (Oakes et al., 2009). This correlates to less total migration and more directional migration on stiffer hydrogels, whereas on soft hydrogels, neutrophils exhibit random walk behavior. When the chemoattractant fMLP is added, neutrophils on stiffer substrates migrate less and more directional than cells on a soft substrate. In another system, neutrophils are perfused through a microfluidic system with a polyacrylamide (PA) hydrogel (Jannat et al., 2010). Similar to the previous example, higher hydrogel stiffness leads to more directional migration, which could be visualized using traction force microscopy (Jannat et al., 2011).

BECs form more vessel-like structures on hydrogels with lower stiffness, whereas more MMPs are produced on higher-stiffness hydrogels (Hanjaya-Putra et al., 2010; Onken et al., 2014). Peripheral blood lymphocytes or neutrophils added to an endothelial monolayer created on soft and stiff hydrogels were found to transmigrate more on more rigid substrates (Onken et al., 2014; Lauridsen and Gonzalez, 2017).

##### Crosslinking/patterning of hydrogel

By specific hydrogel crosslinking, a pattern of altered stiffness can be created. For instance, fibrin hydrogel can be crosslinked with blue light exposure when using a ruthenium crosslinker. A pattern can be created by only exposing specific areas to blue light (Keating et al., 2019). This technique was used to investigate macrophage migration (Hsieh et al., 2019). Again, it was observed that macrophages are flatter and less round on stiff, crosslinked gels compared to soft, not crosslinked gels. In addition, macrophages migrated more on crosslinked hydrogels than non-crosslinked ones, which correlated to increased TNF-α secretion.

##### Topology

Topotaxis is the directional migration of cells across a specific topology. These 3D patterns influence cellular behavior (Matellan et al., 2019). In a study with melanoma cells, it was observed that cells form long filopodia when cultured on areas with fewer pillars. In contrast, cells form short and randomly oriented protrusions in highly dense pillar areas (Park et al., 2016). A study of macrophage migration examined the effect of differently shaped collagen gels (more fibrous or more globular) and found that more macrophages transmigrated across the fibrous collagen compared to the globular collagen (Vasse et al., 2018).

In summary, hydrogel chemistry, composition, porosity, stiffness, crosslinking, and topology play a crucial role in immune cell migration and can affect the *in vitro* modeling of immune cell migration.

#### Experimental aspects of hydrogel ECM creation

Hydrogels are produced from various precursor solutions with different properties. The production and gelation process depends on the hydrogel (Lee et al., 2017). Collagen gelation is both pH- and temperature dependent, and the hydrogel is produced from an acidic precursor solution that is neutralized (Doyle, 2016). GelMA, the modified version of gelatin, is photo-crosslinkable with UV light. Fibrinogen forms a hydrogel upon contact with thrombin within minutes, whereas collagen gelation can take between 30 min and multiple hours, depending on the gelation temperature (Liu et al., 2019). Many hydrogels (e.g., collagen, fibrin, Matrigel) require working on ice during preparation to prevent fast gelation.

The hydrogel properties of different hydrogels also have an effect on pipetting method. Due to the fast gelation time, pipetting of fibrin hydrogels has to be carried out on ice in small volumes that can immediately be transferred from the mixing tube into the chips. On the other hand, collagen gelates slower but the solution is very viscous, hence pipetting of collagen is often carried out with pipette tips with an extra wide opening. These tips are commercially available, but can also be generated in-house by cutting the end off a standard pipette tip.

More information on hydrogel production and gelation, as well as hydrogel incorporation on chip, can be found in these reviews (Caliari and Burdick, 2016; Jiang et al., 2016; Terrell et al., 2020). Depending on the chip design required for the assay, the hydrogel production requirements can play a prominent role in choosing the hydrogel for the organ-on-chip.

One of the key features of hydrogels is their permeability. It can easily be assessed using a permeability assay. A dye coupled to a fluorescent molecule with a known molecular weight (e.g., a RITC-dextran or FITC-dextran) is added, and dye diffusion into the hydrogel is monitored using timelapse microscopy. The permeability can then be calculated based on the following equation (Haase and Kamm, 2017; Ho et al., 2017; Van Duinen et al., 2017):

$$P_{app}=\frac{dC_{ECM}}{dt}\times \left(\frac{V_{ECM}}{A_{EC}\times C_{vessel}}\right)$$ (1)

 P_app_: permeability coefficient.

 C_ECM_: concentration (measured through dye intensity) of the dye in the ECM.

 V_ECM_: volume of the ECM compartment.

 A_EC_: surface of the monolayer where the dye interfaces with the ECM.

 C_Vessel_: concentration (measured through dye intensity) of the dye in the microchannel.

### Microvascular endothelial barrier

Culturing BECs on chip utilizes two main methods to model blood vessels: (1) generating a vessel-like three-dimensional structure and culturing monolayers of BECs on this defined structure (Figure 2) or (2) mixing BECs with mural cells inside a hydrogel to create self-assembled microvessels (Jeon et al., 2015; Zeinali et al., 2018; McMinn et al., 2019; Zeinali et al., 2021).

#### Vascular endothelial cell type affects immune cell behavior

Recent advances in genetic screening, such as single-cell RNA sequencing, have shown that there is not one type but a wide variety of vascular endothelial cell (EC) subtypes specific to the vascular segment and the organ of interest. There are differences in morphology and gene expression between arterial, venous, and capillary BECs without even mentioning lymphatic ECs (Gifre-Renom et al., 2022). Moreover, BECs from different organs function differently, for example, the highly permeable liver sinusoids differ from the tight blood-brain barrier (Gifre-Renom et al., 2022). A human BEC type frequently used in research is the human umbilical vein endothelial cell (HUVEC), as these cells are widely available and easy to obtain from the umbilical cord. Although HUVECs are not microvascular cells, but venous cells, they have allowed for identification of molecular mechanisms of immune cell extravasation. Nevertheless, the choice of endothelial cell type is essential: when comparing a brain microvascular EC line to primary brain microvascular ECs, it was observed that the T Cell diapedesis was lower across primary brain microvascular ECs when compared to the brain microvascular cell line, with higher crawling distance on the primary brain microvascular ECs (Steiner et al., 2011).

#### Experimental aspects of endothelial barrier culture

Culturing BECs seems trivial, but many factors play a role in endothelial function and barrier tightness, such as confluency, cell culture media, culture substrate, shear stress, and perfusion mode.

##### Cell culture substrate affects endothelial cell growth

The choice of culture material influences BEC growth. For example, in a chip with different hydrogels, HUVECs attach and grow nicely on gelatin-gelMA and alginate-gelMA but not on gelatin-alginate (Nie et al., 2018). Similarly, BECs attach more to fibrin hydrogels than to collagen I hydrogels. The most commonly used hydrogels for endothelial cell culture on chip are bovine-derived fibrin and rat tail-derived collagen I, due to their ease of use and low cost.

##### Cell culture media affects endothelial cell function

Cell culture media significantly affects cell morphology and function due to the presence or absence of various soluble factors (growth factors, chemokines, metabolites). For example, HUVECs cultured in M199 media lose their typical morphology only after a few passages, whereas HUVECs cultured in EGM-2 media retain their typical cobblestone morphology for up to 10 passages, most likely due to the additional growth factors present in EGM-2 media (Bala et al., 2011). Specialized media solutions have been developed for specific types of microvascular BECs, or to induce angiogenesis using a pro-angiogenic cocktail (van Duinen et al., 2019).

##### Shear stress and perfusion system

In the human body, BECs experience shear stress from the blood flow. Arterial BECs experience high shear stress, while venous BECs generally experience low shear stress. *In vitro*, shear stress can be applied in microfluidic systems, causing BECs to align along the flow direction and increase barrier tightness (Ohta et al., 2022). When applying alternating flow, this BEC alignment is not observed, indicating that this method of applying shear stress is non-physiological (Lee et al., 2019).

##### Characterization of the endothelial barrier

To experimentally validate if the endothelial barrier is intact and functional, barrier protein expression analysis and permeability assays are important tools. Junctional maturation can be assessed by immunostaining of junctional localisation of VE-cadherin, PECAM-1, JAMs, and possibly other tight junction proteins, to assess the confluency and barrier morphology. On the other hand, a permeability assay quantifies barrier tightness. The permeability of an endothelial monolayer bordering a hydrogel interface can be calculated with Eq. (1). More information on measuring endothelial permeability values in OOCs can be found elsewhere (Haase and Kamm, 2017).

Functionally, it is crucial to know the BEC type in your culture. For example, does this endothelial cell type express and relocate adhesion molecules to the cell membrane upon an infectious stimulus, and does it express known markers for the BEC type of interest? Also, while HUVEC are broadly used for their ease to culture, studies involving tissue-specific BEC are starting to emerge, to take the specificities of these cells into account, for example, in a model of pulmonary infection on chip (Bai et al., 2022). Optimizing the culture conditions mentioned in this chapter can create a favorable BEC culture environment.

### Epithelial barrier

In barrier organs, such as the lung, intestine, and skin, the epithelium is the first physical barrier pathogens encounter upon entry. In the infection-on-chip model, the epithelial barrier is located next to the hydrogel ECM environment and acts as the site of infection (Figure 2).

In the lung, the alveolar epithelium comprises two main cell types: alveolar type I and II cells. The thin type I cells line the alveolar sac and allow gas exchange, covering around 95% of the alveolus (Weibel, 2015). Type II cells, on the other hand, are more stem cell-like, have regenerative potential, and produce surfactant. During infection, alveolar epithelial cells secrete cytokines to activate the endothelial barrier and attract immune cells from the bloodstream (Manicone, 2009). Throughout the pathogen removal process, the epithelium is severely damaged, leading to the accumulation of liquid and cell debris in the alveolar space (Yamada et al., 2016). Multiple lung-on-chip models with an epithelial barrier have been used to study lung infection (Deinhardt-Emmer et al., 2020; Deinhardt-Emmer et al., 2021; Bai et al., 2022).

The intestinal epithelial barrier consists of a multitude of cell types. During infection, the intestinal barrier is disrupted and immune cells can migrate into the underlying tissue. This was shown in a model of T Cell migration across the intestinal epithelial barrier (de Haan et al., 2021).

The skin has a complex, multilayered cellular barrier, that is often modelled in cell culture inserts (Kosten et al., 2016). To model skin toxicity from oral exposure to metals, a multi-organ on chip with gingiva and skin tissue was developed (Koning et al., 2022). In another model, T Cell migration upon skin inflammation was investigated (Ren et al., 2021).

#### Experimental aspects of epithelial cell culture

Epithelial cells can be obtained from primary tissue, but due to limited availability and high variability between donors, on-chip culture of these cells is challenging. In the case of alveolar epithelial cells, alveolar type II cells are highly susceptible to their environment and quickly differentiate to type I cells when placed in culture, leading to a loss of the regenerative type II population. Therefore, cell lines have been used in lung-on-chip models of infection (Huh et al., 2010; Deinhardt-Emmer et al., 2020). However, these cell lines are derived from cancerous tissue and are thus not representing the cells present in normal tissue homeostasis. Similarly, the intestinal epithelium is a complex, self-renewing environment that is often modelled with simple epithelial cell lines on chip (de Haan et al., 2021). However, recent research has shown that intestinal organoids can be grown inside a hydrogel with an *in vivo-*like anatomical structure, leading to a highly *in vivo*-like intestinal model (Nikolaev et al., 2020).

The cell culture media of epithelial cells affects their growth and differentiation. Since alveolar type II cells differentiate in standard culture media, a defined media composition has been developed to maintain alveolar type II cells (Sun et al., 2021). However, not only the epithelial cells are affected by the culture media, but BECs can sense this media too and can migrate from the endothelial compartment towards the pro-angiogenic factors in the epithelial cell compartment.

When co-culturing multiple cell types, optimizing how long cells need to form a functional barrier is necessary. For example, an immortalized alveolar epithelial cell line can take up to 3 weeks to establish a functional barrier, whereas the co-cultured BECs in the model do not need this long culture time to form a functional barrier (Sengupta et al., 2022). Moreover, the air-liquid interface (ALI) culture of pulmonary epithelial cells improves their function (Hiemstra et al., 2019), but a liquid interface is required for chemoattractant perfusion through the epithelial channel. Future studies should look into nebulization of the infectious agent to retain ALI, but this is out of the scope of the current review.

### Immune cells

For modeling the innate immune response on chip, macrophages, monocytes or neutrophils can be cultured inside the OOC (Figure 2; Table 1). The organ of interest plays a significant role in immune cell action, as immune cells are very responsive to the other cells in their environment. Table 1 gives an overview of immune cells used on chip, their source, and isolation method. Both the innate and adaptive immune response have been modeled on chip, or a mixed population of peripheral immune cells was utilized.

**TABLE 1:** Immune cell sources for on-chip culture. This table lists different types of immune cells, their source, isolation method, and examples of chips on which these cells were cultivated. This is a non-exhaustive list of examples of how immune cells were incorporated into OOC models. More methods of immune cell isolation are possible, but have not been tested on chip yet.

Immune cell type	Cell source	Culture/isolation information	Examples of on-chip studies
*Mixed population*			
Peripheral blood mononuclear cells (PBMCs, mixed population)	Human blood-derived	Density gradient separation to obtain PBMCs	Lu et al. (2022)
*Innate immune response*			
Macrophages	Mouse bone marrow	Isolation from bone marrow, differentiation with M-CSF	Li et al. (2018), Thacker et al. (2020)
	RAW264.7	Cell line	Huang et al. (2009), Zervantonakis et al. (2012)
	THP-1	Monocyte cell line, can be differentiated with PMA	Sharifi et al. (2019), Yin et al. (2019), Gjorevski et al. (2020), Pérez-Rodríguez et al. (2022)
	Human blood-derived	Density gradient separation to obtain PBMCs followed by monocyte selection and differentiation	Deinhardt-Emmer et al. (2020)
Neutrophils	HL-60	Cell line	Han et al. (2012), Chen et al. (2018)
	Human blood-derived (1)	Density gradient separation	Jannat et al. (2010), Han et al. (2012), Wu et al. (2015), Yang et al. (2017), McMinn et al. (2019), Gjorevski et al. (2020), Riddle et al. (2022)
	Human blood-derived (2)	Neutrophil isolation with microfluidics	Hamza and Irimia (2015), Wang and Irimia (2018)
*Adaptive immune response*			
Dendritic cells	Mouse bone marrow	Isolation from bone marrow and differentiation *in vitro*	Haessler et al. (2011), Aizel et al. (2017)
	MutuDC	Cell line	Moura Rosa et al. (2016)
	Human blood-derived	Density gradient separation to obtain PBMCs followed by cell differentiation	Parlato et al. (2017)
T Cells	Human blood-derived (1)	Density gradient separation to obtain PBMCs followed by T Cell selection	de Haan et al. (2021), Van Steen et al. (2021)
	Human blood-derived (2)	T and B Cell isolation with microfluidics	Chiu et al. (2019)

Even though the most physiological model would contain only freshly isolated human immune cells, the choice of cells also highly depends on the availability of specific cell types, the research question, and the organ of interest (Granton et al., 2018). Generally, combining multiple immune cell types to investigate their role during infection would be interesting, for example, by combining tissue-resident macrophages in the ECM hydrogel compartment with monocytes in the vascular compartment (Chen et al., 2023). In the infection-on-chip model, the main focus is the extravasation and migration of peripheral immune cells perfused through the blood vessel (Figure 2).

#### Experimental aspects of immune cell culture on chip

Isolation of human peripheral immune cells is traditionally carried out by gradient separation from whole blood or buffy coat, with post-processing of the cells to obtain specific populations, such as differentiated macrophages (Grievink et al., 2016; Golke et al., 2022). Recently, negative and positive selection-based methods with magnetic sorting have been developed (Chometon et al., 2020). These methods, although more costly than traditional gradient separation, achieve a higher purity of the immune cell subset of interest. Depending on the immune cell type of interest, different isolation methods can be elected (Table 1).

##### Culture conditions influence immune cells

Like endothelial and epithelial cells, immune cells also react to their cell culture media. For example, the foreign material in fetal bovine serum (FBS) can cause monocyte activation. Hence, human autologous serum is used for immune cell culture to prevent activation (Deinhardt-Emmer et al., 2020). To visualize immune cell migration, fluorescent labelling of immune cells is standard practice. However, this chemical labeling can activate immune cells or when membrane labeling is used, limit their migration by inhibiting cell membrane motility, which is why one should label the cytoplasm or genetically modify the cells. Thus, the correct labelling solution should be used, and immune cell activation should be checked. Methods to check for immune cell activation are flow cytometry, microscopy, or assessing cytokine secretion to measure activation markers.

##### Shear stress on immune cells

In the human body, immune cells in the bloodstream experience different levels of shear stress. A majority of *in vitro* research is carried out statically, without the perfusion of immune cells (Huang et al., 2009; Han et al., 2012; Wu et al., 2015; Aizel et al., 2017; Parlato et al., 2017; McMinn et al., 2019). For a more physiological model, immune cells should be perfused through the blood vessel channel to observe rolling, arrest, polarization, crawling and finally diapedesis across the microvascular endothelial layer into the hydrogel (Bianchi et al., 2013). Adding perfusion has a significant effect on immune cell migration in general, as shown in a study where macrophages embedded inside a hydrogel were exposed to interstitial flow or kept under static conditions (Li et al., 2018). In this model, interstitial flow increased macrophage migration inside the hydrogel. Furthermore, adding tumor cells showed that both interstitial flow and the presence of tumor cells increase macrophage motility (Lee et al., 2020). In another study, perfusing neutrophils through a microfluidic channel increased the number of neutrophils migrating into a collagen matrix towards tumor spheroids, compared to keeping the neutrophils static (Surendran et al., 2021). Additionally, T Cells, but not neutrophils, can migrate against the physiological flow in an *in vitro* flow chamber coated with ICAM-1 and SDF-1 or fMLP (Valignat et al., 2013). Physiological shear stress values between 2–12 dyne/cm^2^ were compared, and the higher the shear stress, the straighter the cells moved along or against the flow direction. Under low shear stress, more random walk behavior was observed. Although the flow rate influences migration direction, the migration speed was not affected, which was confirmed in another study (Rainger et al., 1999). Overall, these results indicate that shear stress impacts immune cell migration, showing increased migration capacity of immune cells under physiological fluid flow.

##### Cell-cell interactions

Next to the interactions with the microenvironment, cell-cell interaction is crucial for immune cell function. In a study of macrophage migration within hydrogels, co-culture with fibroblasts increased macrophage migration and led to tunnel formation (Ford et al., 2019). Hydrogel stiffness only decreased in cocultures of fibroblasts and macrophages, and remained unaltered in monocultures. Moreover, macrophages differentiated more towards an M2 phenotype in co-culture, whereas in monoculture, a mixed M1/M2 population is observed. In a study of neutrophil migration, in the presence of an IL-8 gradient, neutrophils do not migrate much, but with an endothelial layer between the neutrophils and the IL-8 gradient, neutrophil migration is significantly increased (McMinn et al., 2019). In a lymph node-on-chip system, naïve T Cells were exposed to antigen-presenting dendritic cells and binding was analyzed, showing interactions between different types of immune cells (Chiu et al., 2019). Generally, cell-cell interaction influences immune cell behavior and is an important consideration when testing immune cell migration on chip.

### Inflammation or infection

To attract innate immune cells, a chemical signal to direct them is necessary. This chapter focuses on solutions to generate a chemotactic gradient for immune cell migration. First, it should be mentioned that chemotaxis and haptotaxis are different processes that both take place *in-vivo*. Chemotaxis is defined as the directional migration of immune cells following a gradient of soluble chemokines in the environment, released from the site of infection (Petri and Sanz, 2018), while haptotaxis describes the migration of immune cells induced by a gradient of ECM-bound chemokines within the microenvironment. Here, the focus will be on chemotaxis, however, with ECM-based hydrogels, haptotaxis can also occur.

Many chemotactic agents are available, such as bacteria or viruses, factors secreted by other cells cultured in the system, or factors added to the cell culture media. For example, in an OOC, neutrophil extravasation can be induced by the presence of bacteria, pre-activated BECs, or a gradient of the bacterial peptide fMLP (Han et al., 2012; Hind et al., 2021). Here, we discuss forming a chemotactic gradient in a microfluidic device and some common chemotactic agents used for attracting immune cells.

#### Forming a chemotactic gradient

The formation of a chemotactic gradient is crucial to investigate immune cell migration. In the absence of a gradient, when fMLP concentration is uniform, neutrophils migrate randomly in an inflammation-on-chip model using a polyacrylamide (PA) hydrogel. However, when an fMLP gradient is established, the cells move towards the higher concentration of the gradient (Jannat et al., 2010). The dominating transport mechanism in static microfluidic systems is diffusion, the random movement of particles inside a space, which eventually leads to an even spread of these particles (Figure 2). Diffusion is driven by a concentration difference of particles within a given space and is described by Fick’s first and second laws of diffusion (Figure 2). It is important to realize that the diffusion coefficient depends on the viscosity, which can vary importantly from one hydrogel to the other. It is thus essential to measure the hydrogel viscosity used and adjust the gradient’s setup accordingly. Moreover, the environment temperature and the chemoattractant’s particle size should be accounted for as well.

Many microfluidic systems use diffusion to create a chemotactic gradient (Zhao et al., 2020). A gradient can be created by simply pipetting the chemoattractant next to the hydrogel and letting it diffuse through the hydrogel or by using a pipette tip as chemottractant reservoir that is placed in one corner of the hydrogel and slowly releasing the chemoattractant, which can then diffuse through the hydrogel (Oakes et al., 2009). The drawback of using these basic gradients is that the diffusion flux (as shown in Figure 2) will decrease until the concentration is uniform throughout the entire space. To avoid this limitation, one can perfuse the chemoattractant continuously in the epithelial channel (source channel, Figure 2), while perfusing cell culture medium without chemoattractant in the endothelial channel (sink channel, Figure 2). Two main methods aimed at perfusing microfluidic channels are commonly used: pressure-driven and flow-controlled pumping systems. Tilters, also called rocking platforms, are broadly used pressure-driven systems for their simplicity as they do not require any tubings. Piston-pumps and peristaltic pumps are typical flow-controlled pumping systems.

#### Perfusion in microfluidic devices

##### Pressure controlled pumping systems

**FIGURE 5:**
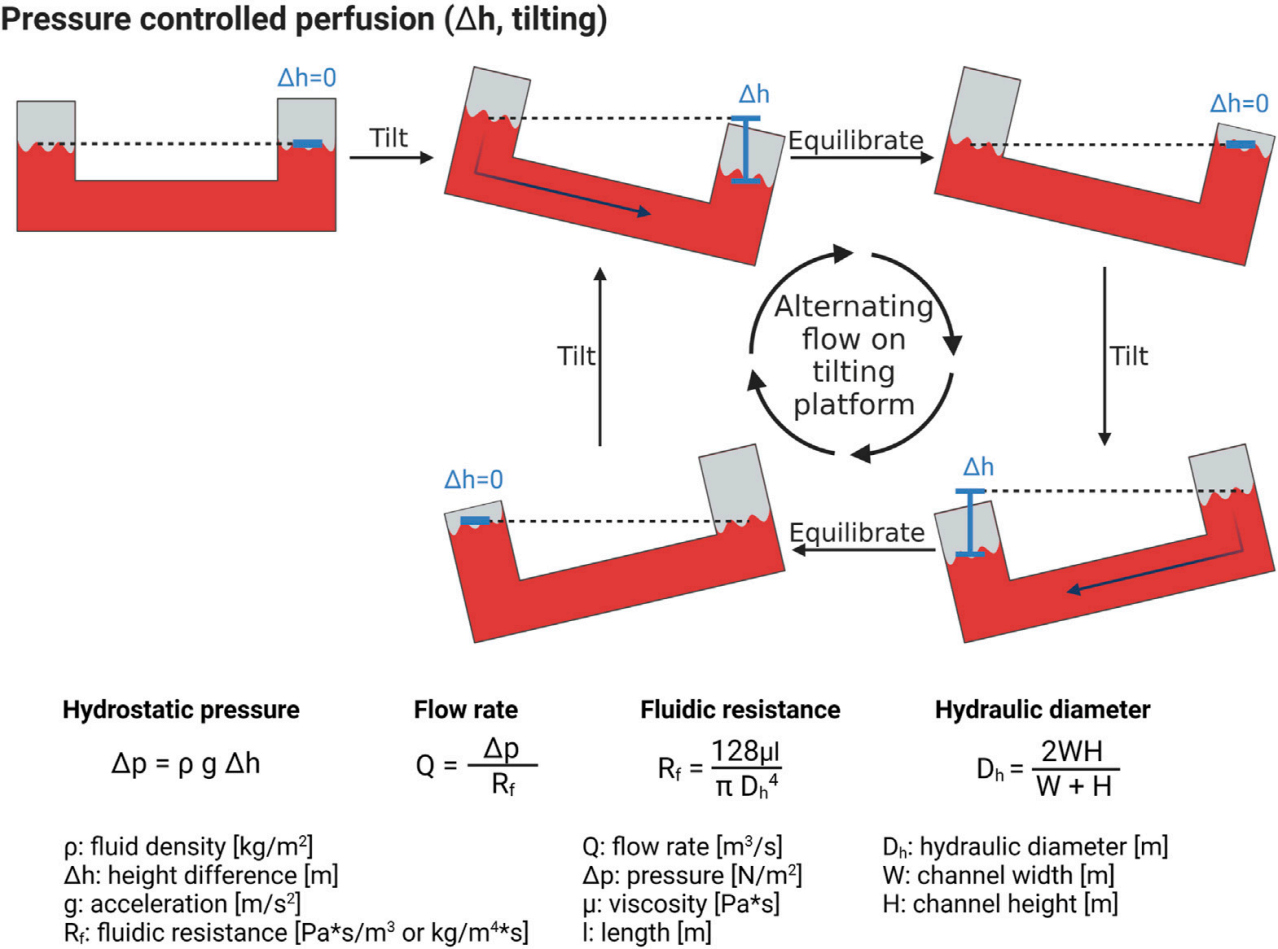
Hydrostatic pressure-induced perfusion of microfluidic systems. By tilting the microfluidic chip, a height difference in the liquid reservoirs is established, with a correlated difference in pressure, leading to fluid flow. Formulas to calculate the flow rate inside a tilting microfluidic system are presented. Created with BioRender.com.

A simple yet effective method to create flow inside a microfluidic system is by applying a pressure in a closed reservoir connected to a microfluidic channel (Luo et al., 2009). The pressure acting on the liquid contained in the reservoir induces a flow rate in the channel according to Poiseuille law. The flow rate is a linear function of the applied pressure and is limited by the fluidic resistance of the microfluidic channel (equations in Figure 5). The fluidic resistance of channels with rectangular cross-sections that are typical in microfluidics, can be calculated using the hydraulic diameter approximation. To avoid the need of tubing (de Graaf et al., 2022) to pressurize the reservoir, a hydrostatic pressure difference between the inlet and the outlet reservoirs can simply be used. One popular method that uses this approach is tilting platforms, also called rockers. They do not need much space and can thus be implemented for higher thoughput solutions (Luo et al., 2009; Yang et al., 2017; Li et al., 2018). A tilter is illustrated in Figure 5, in which the fluid level in the two reservoirs equilibrate with time. Once the levels are identical, the platform is tilted in the opposite direction, resulting in an alternating (bi-directional) flow with variable flow rate (Kim et al., 2015; Poussin et al., 2020; de Haan et al., 2021).

Tilter platforms have some drawbacks, such as the limited range of flow rates than can be generated, which depends on both the platform’s tilting angle and the channel geometry. In addition, the flow is not constant and cannot be controlled. For instance, when an air bubble or cells obstruct the channel, the fluidic resistance increases, resulting in a decrease in flow rate. Finally, alternating flows are non-physiological. Recently, more evolved microfluidic systems allow for a uni-directional flow with tilter platforms (Wang and Shuler, 2018).

##### Controlled flow pumping systems

In contrast to pressure driven flow, controlled flows can be created by piston-pumps or peristaltic pumps, which can generate constant and uni-directional flow (Huh et al., 2010; Moura Rosa et al., 2016; Menon et al., 2017). In contrast to peristaltic pumps, piston pumps do not mechanically stress the cells, however, cells might sediment inside the piston or syringe. Peristaltic pumps do not present this limitation, however may induce hemolysis and/or cell activation due to the mechanical forces exerted on the cells (Hajipouran Benam et al., 2016; Sharifi et al., 2019).

#### Practical aspects of inducing infection

##### Perfusion

While pumps are very accurate and are a good option for perfusion, there are some disadvantages to consider. First, the tubing must be sterilized and kept sterile throughout the experiment. Second, there is a high risk of bubble formation inside the tubing, which could damage the cell culture. To prevent bubbles in the OOC, bubble traps have been developed, and all media/buffers and tubing are placed inside the incubator to prevent bubble formation through changes in the humidity and temperature. Thirdly, connecting multiple OOCs to a pump can be cumbersome and time-consuming, leading to lower experimental throughput. A final consideration is the pump location: when the pump is placed inside the incubator, it is close to the chips, and the tubing can be short. However, this can cause overheating of the incubator, pose sterility issues, and the humid environment is detrimental to the pump. The pump can also be placed outside the incubator, increasing the tubing length required and, thus, the system’s complexity, especially if multiple OOCs are connected. Overall, pumps offer a reliable method to apply shear stress to an OOC, but throughput is decreased.

##### Chemotactic agents: choices and considerations

Chemotactic agents can be subdivided into two main categories: chemoattractant molecules, and living agents or pathogens. Importantly, when using a chemoattractant molecule, *inflammation* is modelled on chip, and only when adding a pathogen, *infection* is modelled. Of course, an infection will also cause inflammation on chip. To attract immune cells, different types of chemotactic molecules can be used. For example, these can be cytokines normally produced by other cells in the environment after infection, such as IL-2 or IL-8 (Irimia et al., 2006; Han et al., 2012; Wu et al., 2015). Pathogen-specific particles can also be used as a chemoattractant. Here, an infection with a pathogen is mimicked by inducing inflammation without needing to cultivate actual pathogens and apply the safety restrictions that accompany the use of dangerous pathogens. Some generally used molecules are PolyI:C, a viral mimic; lipopolysaccharides (LPS), a bacterial membrane saccharide; and fMLP, an immune cell-binding molecule secreted by bacteria (Jones et al., 2014; Hajipouran Benam et al., 2016; Chandrasekaran et al., 2017; Wang and Irimia, 2018). Lastly, exposure to nanoparticles can also lead to immune cell activation and migration. For all these chemotactic agents, it is crucial to choose the right concentration in which immune cells are activated but not overstimulated.

To model infection, viruses, fungi, or bacteria can be added to the infection-on-chip model. Because bacteria are still alive, interactions between the bacteria and the immune cells can be studied, which was already reported with *E. Coli* and tuberculosis bacteria (Huh et al., 2010; Thacker et al., 2020). Similarly, inhibiting fungal growth on chip through a neutrophil response can be investigated (Barkal et al., 2017). Various organ-on-chip systems of SARS-CoV2 infection and other viral infections have been recently developed, as described in other reviews (Chakraborty et al., 2020; Tang et al., 2020). The interactions between viruses, bacteria, and immune cells can also be studied, such as in a lung-on-chip with a double influenza and *Staphylococcus aureus* infection (Deinhardt-Emmer et al., 2020). Using live bacteria or viruses is more clinically relevant than a synthetic mimic but also poses more danger to the researcher handling these agents. Thus, appropriate safety measures have to be taken. One can also use cells to produce the chemoattractant within the system. For example, tumor cells secrete various agents that can attract dendritic cells, neutrophils, and macrophages (Huang et al., 2009; Parlato et al., 2017; Lee et al., 2021).

Overall, there are several options to model infection or create a chemotactic gradient, varying in complexity and type of infection.

### Readouts of migration

**FIGURE 6:**
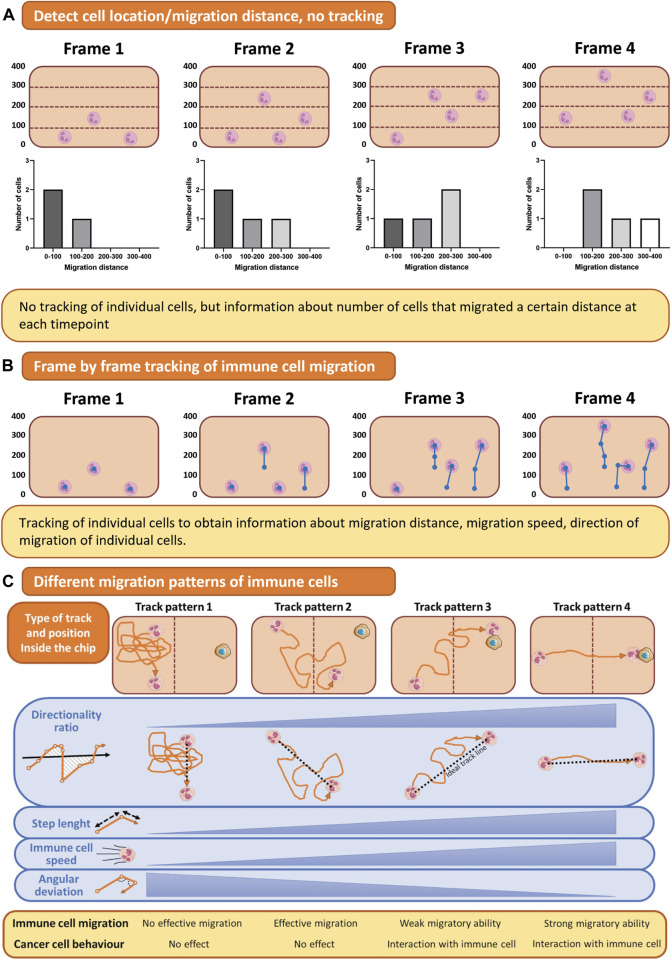
Quantification of immune cell migration. **(A)** For assessing immune cell location, the number of cells in a predefined area can be counted automatically. **(B)** Timelapse imaging of migrating immune cells results in a collection of images in which immune cells can be traced. **(C)** Tracking of immune cell migration shows different track patterns from which directionality, speed, and step length can be analyzed. This figure shows tracks of a single cell as an example for simple visualization purposes, but multiple cells can be tracked using these methods. Figure C was adapted from Mattei et al. (2021), under the terms of the Creative Commons Attribution License(CC BY). (https://creativecommons.org/licenses/by/4.0/).

To assess immune cell migration during infection, microscopy, specifically live cell imaging, is an essential tool. Accurate visualization of immune cell migration can be obtained with live cell imaging, where timelapse intervals and total imaging time are crucial to capture the process of interest (Figure 6A). However, there must be a balance between avoiding phototoxicity and increasing imaging frequency and length to capture all processes of interest.

Not only the microscopy itself but also the chip design influences the quality of the imaging and subsequent data analysis. For example, in the first lung on chip published by Huh et al. (Huh et al., 2010), neutrophils were migrating towards the site of infection, but migration dynamics could not be quantified, as it was occurring vertically across a porous membrane, and the immune cell migrated out of focus. Thus, to fully image the extravasation process, generating a model where immune cells migrate in a horizontal plane instead of vertically across a membrane is essential, as exemplified by multiple experimental designs (de Haan et al., 2021; Pérez-Rodríguez et al., 2022; Riddle et al., 2022). Live imaging of one focal plane can be carried out, and immune cell migration can be tracked.

After a timelapse video has been created, analysis of migration distance, speed, and direction is the next challenge (Figure 6). The authors refer to the following review for a more detailed overview of which parameters can be measured from a live imaging dataset of immune cell migration (Mattei et al., 2021). Traditional image analysis software such as ImageJ/FIJI or Imaris can aid in cell tracking, but these semi-automated solutions do not work for all imaged time-lapses. Thus, a tracking algorithm to track immune cell migration on chip can be developed in-house, for example, with Matlab (Pérez-Rodríguez et al., 2022). To simplify the analysis, an alternative to tracking individual immune cells is quantifying immune cells inside the hydrogel and their distance from the endothelial barrier over time (Figure 6B). With this method, no decisions on cell tracking are made, making the analysis less detailed but more robust. Next to imaging and tracking immune cells migrating horizontally in one focal plane, recent advances in spinning disk confocal microscopy have allowed for 3D timelapse imaging, to track cellular migration in all directions (Wen et al., 2021). This technique has not been applied in OOC models of infection yet, but would be of interest.

Advances in artificial intelligence (AI) have enhanced analysis methods, with the potential for more automation of (tracking) data analysis in the future (Ren et al., 2022). The first microfluidic experiments with deep learning algorithms to track cells have already been performed, such as an analysis pipeline to track bacteria in a microfluidic chip (Lugagne et al., 2020) and an algorithm to track tumor cell migration on chip (Zhang et al., 2018). In the second study, the migration direction and speed of the cells could be analyzed. So far, this technology is mainly used on relatively simple, single-cell OOC models and relies on manual adaptation during training (Ren et al., 2022). However, with more and more data becoming available, deep learning algorithms provide an immense opportunity for future analysis of immune cell migration on chip.

## Future perspectives

### Engineering the next-generation organ-on-chip models

Current models of infection on chip have the common drawbacks of most OOC models, including only low to medium throughput, using the highly absorbing material PDMS for molding structures, and a lack of translation to clinical data. These issues must be addressed to engineer the next-generation of OOC models. To increase fabrication throughput and replace PDMS, OOCs have been fabricated with injection molding of polystyrene or similar transparent polymers, for example, to fabricate a high throughput OOC of cancer spheroid vascularization (Kim et al., 2022). Comparison of the data generated with OOCs to previously acquired data from animal studies and clinical trials is ongoing. For example, in the field of infection, many studies of immune cell extravasation have been carried out in mouse, rat, and rabbit models. With the appearance of new OOC models, it is important to investigate how these data can be compared and how both animal and OOC models translate to human diseases.

### Towards *in vivo* complexity

Additional complexity can be added to increase the relevance of OOC models of infection even further, for instance by mimicking both the innate and adaptive immune responses. This and other improvements may include replacing cell culture media with whole blood, adding tissue-resident immune cells, and integrating the lymphatic system.

Currently, only a subset of immune cells is perfused into a culture media or a buffer, but whole blood could be perfused to increase relevance. Not only does whole blood contain all cell types of interest, but blood also has a different viscosity and, therefore a different effect on the endothelium. Preliminary tests with whole blood perfusion in OOCs have been carried out and appear promising (Menon et al., 2017; Golomingi et al., 2022). Microfluidic devices can be modified to directly use whole blood for selective migration of neutrophils toward a site of infection. For example, whole blood was added in a microfluidic device with infected skin tissues, and neutrophil migration towards the infected skin tissues was observed (Kim et al., 2019b).

Another important part of immune surveillance is the tissue-resident immune cell population. These cells, mainly tissue-resident macrophages and T Cells, have a distinct gene expression profile and monitor the tissue microenvironment. Since these cells are present inside the tissue and their numbers are low, isolation is challenging, and source material is scarce. Further research is necessary to understand how these cells can be best isolated and cultured *in vitro*.

Lastly, a crucial part of the immune system, the lymphatics, is often overlooked. Generally, the main focus of infection-on-chip models is the interaction between immune cells circulating in blood vessels and the infected tissue, but for activation of the adaptive immune response, migration of dendritic cells from the site of infection to the lymph nodes to activate antigen-specific T Cells is necessary. Individual systems modeling lymph nodes or vessels on chip have been developed (Selahi et al., 2021; Shanti et al., 2021), however, to our knowledge, lymph vessels have not been incorporated into an OOC model of vasculature or other organs.

### Cancer-immune cell interactions: Immunotherapy

Next to infection, immune cells are also involved in other conditions, such as cancer, which adds the opportunity to utilize immunocompetent OOC models not only to model infection but also to model cancer immunotherapy. Using the human body’s natural defense system, cancer immunotherapy targets tumor tissue with the immune system, especially T Cells. Multiple studies are emerging with microfluidic models of immune cell migration towards a tumor, with known and unknown immunotherapy drugs enhancing tumor suppression by T Cells (Pavesi et al., 2017; Deng et al., 2018).

### Correlating OOC data to the clinics: towards clinical relevance of OOC models

This review has discussed the generation of an infection-on-chip model to investigate infectious processes in a 3D *in vitro* model. By incorporating various cell types, cultured on or in hydrogel ECM environments, and perfusing the endothelial vessels, OOC models provide a more sophisticated alternative to basic 2D *in vitro* cultures. The modularity and versatility of OOC models enable the study of (disease) mechanisms in the presence or absence of particular aspects of the system. For example, research has demonstrated that the lack of endothelial cells prevents the migration of immune cells into a hydrogel, whereas the presence of endothelial cells significantly increases the number of immune cells that migrate (Wu et al., 2015; McMinn et al., 2019). It is impossible to study the impact of endothelium on immune cell migration *in vivo* as it is not feasible to completely eliminate endothelial cells from an animal. Consequently, OOC models offer an opportunity to supplement the findings from animal studies.

However, the challenge remains in translating the data obtained from these complex models to human disease. Initially, OOC models aim to replicate the disease symptoms observed in the clinic, such as drug-induced pulmonary oedema (Huh et al., 2012). Moreover, during the COVID-19 pandemic, a list of antivirals were tested in a lung-on-chip, showing that hydroxychloroquine, a drug that demonstrates efficacy against SARS-CoV2 in cell lines, did not exhibit antiviral effect on chip (Si et al., 2021). This finding translated to the clinic, where this drug proved ineffective against SARS-CoV2. Thus, to establish a correlation between research findings on infection-on-chip models and clinical data, clinical observations and readouts must be shown on chip.

## Conclusion/summary

Overall, the OOC field continues to develop at a fast pace with innovations occuring regularly. With new chip materials, the addition of AI, and the integration of different types of primary cells, these models will continue to evolve in the future.

The constant enhancement of OOC models aims to replicate human (patho)physiology with greater precision. In this review, attention was directed towards a particular category of OOC models that imitate the innate immune cell extravasation process during infection by means of a hydrogel ECM. To model an innate immune response on chip, the following main components are needed: the barrier (microvascular endothelium, hydrogel ECM, epithelium), the (perfused) immune cell, and the chemotactic gradient mimicking the infection. With this tutorial review, practical experience for designing an infection-on-chip experiment has been summarized to encourage the further development of these models in research institutions worldwide.

## Abbreviations

AI, Artificial Intelligence; APTES, (3-aminopropyl)triethoxy silane; EC, Endothelial Cell; ECM, Extracellular Matrix; HA, Hyaluronic Acid; HUVEC, Human Umbilical Vein Endothelial Cell; MMP, Matrix Metalloproteinase; OOC, Organs-on-chip; PA, Polyacrylamide; PAMP, Pathogen-Associated Molecular Pattern; PDMS, Polydimethylsiloxane; PEG, Polyethylene Glycol; PRR, Pattern Recognition Receptor.
